# Editorial: Systems biology and antimicrobial drug resistance

**DOI:** 10.3389/fmicb.2024.1481911

**Published:** 2024-09-02

**Authors:** Vijay Soni, Aditya Kumar Sharma, Neha Dubey, Saurabh Mishra

**Affiliations:** ^1^Division of Infectious Diseases, Weill Department of Medicine, Weill Cornell Medicine, New York, NY, United States; ^2^Department of Pathology, College of Medicine, University of Illinois at Chicago, Chicago, IL, United States; ^3^School of Biological and Life Sciences, Galgotias University, Gautam Budh Nagar, Uttar Pradesh, India; ^4^Department of Molecular Microbiology, Center for Women's Infectious Disease Research, Washington University School of Medicine, St. Louis, MO, United States; ^5^Department of Microbiology and Immunology, Weill Cornell Medicine, New York, NY, United States

**Keywords:** antimicrobial resistance (AMR), system biology, metabolomics, omics, genomics, proteomics, transcriptomics

**Figure 1:**
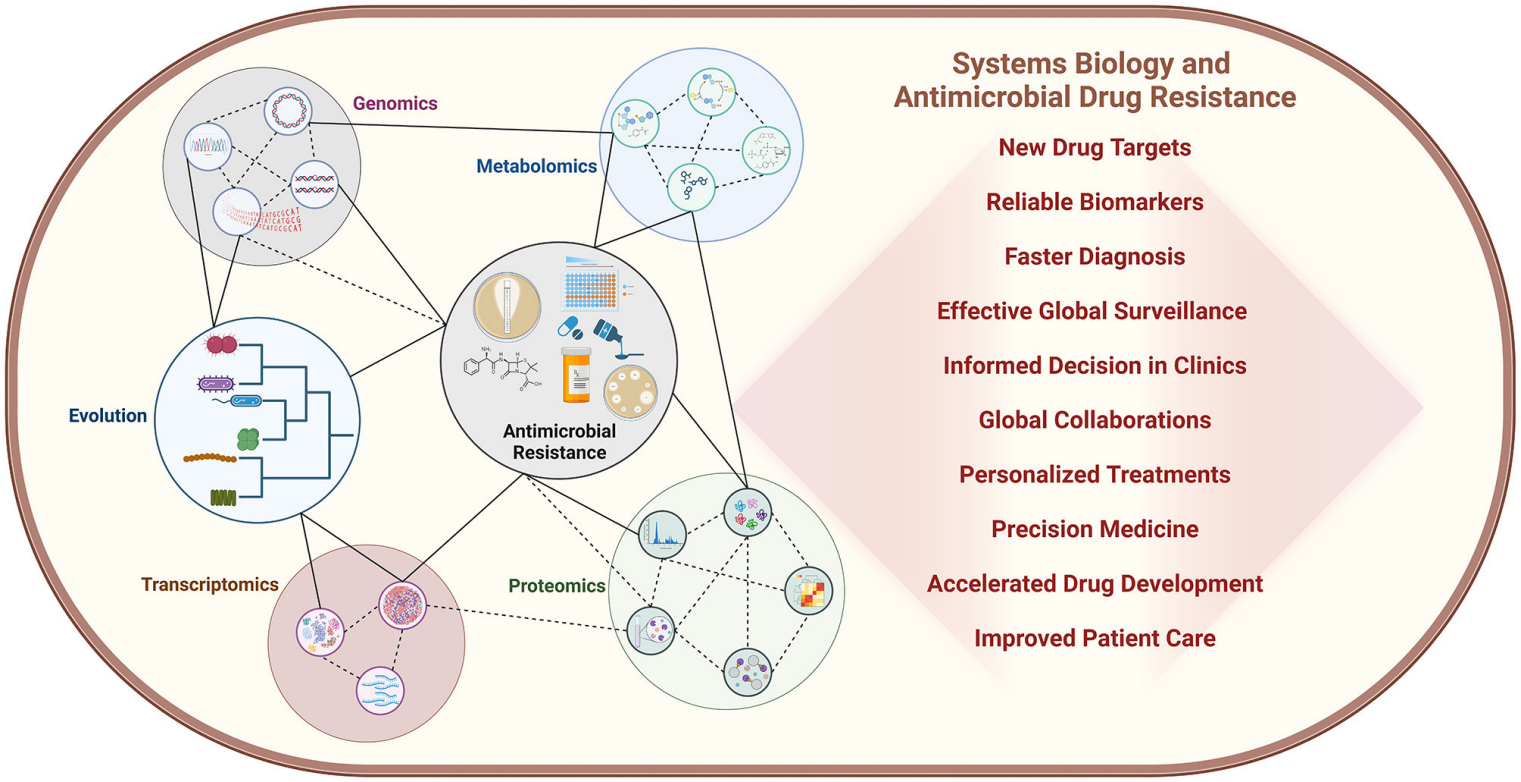
Systems biology is playing a crucial role in elucidating bacterial physiology and the development of antimicrobial resistance, revealing complex molecular mechanisms and their interconnected relationships at a systems level. The impact of this field is profound, with far-reaching implications for both clinical practice and research laboratories. Moreover, the integration of artificial intelligence and machine learning technologies is rapidly accelerating progress, unlocking new insights and innovations in the fight against AMR.

For thousands of years, bacterial infections have been the primary cause of mortality. The discovery of antibiotics offered a temporary solution to this crisis, but their effectiveness was short-lived due to the remarkable adaptability and rapid evolutionary strategies employed by bacterial pathogens. As a result, the emergence of antimicrobial resistance (AMR) has become an even more pressing concern, posing a significant threat to global health. With the advent of novel high throughput molecular biology technologies, systems biology has emerged as a powerful tool to study AMR by integrating data from different methods, including genomics, transcriptomics, proteomics, and metabolomics (Figure 1). It is helping to identify novel biomarkers and targets for faster diagnosis and efficient drug development (Figure 1). Considering all these facts we have edited the Research Topic “*Systems Biology and Antimicrobial Drug Resistance*” to cover research articles highlighting the critical issue of bacterial AMR and explore the potential of systems biology in identifying new drug targets and combatting AMR, particularly in *Mycobacterium tuberculosis, Acinetobacter* spp., *Staphylococcus aureus*, and *Pseudomonas aeruginosa* infections where only a limited number of antibiotics are available for treatment.

Starting from clinical study from Wu et al. examined the clinical, pathological, and drug-resistant characteristics of patients with spinal and pulmonary tuberculosis. Spinal tuberculosis exhibited a similar pathology of chronic granulomatous inflammation and caseous necrosis. However, the spinal tuberculosis group demonstrated higher amounts of granulation tissue, abscess, acute inflammation, caseous necrosis, and exudation. MRI findings also revealed paravertebral and bilateral psoas muscle abscesses, surrounding soft tissue edema, and infectious vertebral body lesions. In the spinal tuberculosis group, drug resistance analysis revealed higher incidences of resistance to rifampicin (RFP) + isoniazid (INH) + ethambutol (EMB) and RFP + EMB, but lower incidences of resistance to RFP + INH + STR and INH + EMB.

In another clinical work, Hou et al. analyzed Methicillin-resistant *Staphylococcus aureus* (MRSA) strains from a hospital setting in China. Their work is focused on prevalence, antibiotic resistance, and the genomic characteristics of the bacteria. Among

212 collected strains, 38 were identified as MRSA and subjected to further analysis. These MRSA strains showed resistance to penicillin, erythromycin, and clindamycin but remained sensitive to vancomycin and linezolid. The study also revealed a high detection rate of biofilm genes within the MRSA strains. Notably, the ST59-SCCmecIV-t437 clone exhibited a higher prevalence of resistance genes and a stronger biofilm-forming capacity. Conversely, the ST59-SCCmecV-t437 clone displayed a higher positive rate for the *pvl* gene, leading to increased pathogenicity and a greater risk of causing systemic infections.

Similarly, Miao et al. investigated the prevalence, genetic makeup, and antibiotic resistance patterns of *Streptococcus pneumoniae* in 263 pneumococcal disease patients at West China Hospital. They revealed 19F as the predominant serotype. Penicillin resistance was notably high, affecting 82.35% of meningitis and 1.22% of non-meningitis cases. The prevalence of *ermB* and *tetM* resistance genes was substantial, correlating with high levels of erythromycin and tetracycline resistance respectively. Interestingly, all isolates remained susceptible to vancomycin and linezolid.

Further, in a related article, Handa et al. investigated the resistance patterns associated with in and outpatients from a tertiary care hospital. This study aimed to identify the resistance patterns of several pathogenic bacteria in a healthcare setting. The investigation encompassed screening nearly ~450 bacterial strains isolated from over ~900 patients through a wide antibiotic susceptibility test screen. Their research has contributed to identifying effective drugs for controlling infections caused by AMR bacteria in the population and for monitoring the prevalence of these drug-resistant bacterial pathogens. Understanding these patterns is crucial for guiding effective treatment strategies toward the challenges associated with the anti-microbial resistance.

Toward understanding the mechanism of AMR, Huangwei et al. investigated mechanisms underlying multi-drug resistance (MDR) in the fungus *Clarireedia jacksonii* by analyzing isolates LT15 and LT586. Their study revealed that LT586 exhibited resistance to three fungicides: iprodione, propiconazole, and boscalid. Using RNA-seq analysis, they found significant differential gene expression between MDR LT586 isolate and the fungicide-sensitive LT15 isolate. LT586 exhibited downregulation of 306 genes involved in metabolic pathways such as butanoate metabolism, terpenoid backbone biosynthesis, tyrosine metabolism, and starch and sucrose metabolism. Conversely, 153 genes were upregulated in LT586, with enrichment of different functional categories including peroxisome function, glycerophospholipid metabolism, protein processing in the endoplasmic reticulum, and ABC transporters. This differential gene expression pattern suggests metabolic reprogramming and enhanced efflux mechanisms as contributing factors to the development of the MDR phenotype in LT586.

Further, Shah et al. performed a proteomics-based analysis to identify novel target proteins in drug-resistant *Prevotella melaninogenica*. The study yielded 1,415 core proteins from 14 complete genome sequences of *P. melaninogenica*. Using subtractive proteomics, reverse vaccinology techniques, and rigorous bioinformatics analysis, 18 candidate proteins were selected. Two of these candidates, ADK95685.1 and ADK97014.1 were chosen for the design of a multi-epitope vaccine against *P. melaninogenica* infection.

In the same direction, Qin et al. have identified the *htpG* gene as a critical factor in antibiotic resistance development in *Vibrio mimicus* SCCF01. Deletion of the *htpG* gene led to increased lipopolysaccharide production, decreased levels of glycerophospholipids, and weakened efflux pump activity. Moreover, the *htpG* deletion strain became less susceptible to β-lactam antibiotics due to impaired peptidoglycan synthesis and disrupted peptidoglycan recycling and regulation.

Additionally, in a related context, research publication from Qiu et al. investigates the antibiotic resistance mechanisms of carbapenem-resistant *Acinetobacter baumannii* (*A. baumannii*) clinical isolates from Guiyang, China. This study enhances the broader understanding of AMR by offering detailed molecular and genetic insights into the mechanisms of carbapenem resistance in *A. baumannii*, a critical nosocomial pathogen. Authors used the combination of multilocus sequence typing (MLST) and transcriptome analysis to identify the genes enriched in the AMR strains of *A. baumannii* isolates. The resistance locus was identified in the genes associated with key bacterial processes including efflux pumps, biofilm formation, and cell wall processes. This provides crucial information for formulating more effective strategies to combat AMR in clinical settings.

Subsequently, the study by Zhu H.-Y. et al. uncovers a key molecular mechanism in the bacteria, *Weissella cibaria* (*W. cibaria*), a bacteria that persists under ciprofloxacin stress. Authors unveiled the molecular mechanism of the PemIK toxin-anti-toxin system, that contributes to the antibiotic resistance behavior of the *W. cibaria* by aiding in the bacterial transition between the actively replicating and persistent state. The endonuclease activity of the toxin PemK of the PemIK system targets the mRNA-encoding enzymes involved in glycolysis, the TCA cycle, and the respiratory chain pathway. This metabolic disruption leads to a decrease in ATP levels in the bacterial cells, and an increase in persister frequency. The authors also identified Arg24 residue of PemK as crucial for its activity. This system allows *W. cibaria* to survive ciprofloxacin treatment not through genetic resistance, but through a phenotypic persistence state. This persistence mechanism can contribute to treatment failures and recalcitrant infections, as a subpopulation of bacteria can survive antibiotic exposure and repopulate once treatment stops.

In continuity, Zhu L. et al. demonstrated the importance of the ISKpn element in conferring *mgrB* gene mutations in ST11 hypervirulent colistin-resistant *Klebsiella pneumonia*. They identified three IS elements inserted themselves into or near *mgrB* gene that confer resistance. These elements are ISKpn26, ISKpn14, and IS903B. Additionally, analyzed isolates exhibited high resistance to multiple antibiotics including TCC (Ticarcillin), TZP (Piperacillin/Tazobactam), CAZ (Ceftazidime), and COL (Colistin), and a significant portion, 85%, displayed resistance to both DOX (Doxycycline) and TOB (Tobramycin). Further analysis identified these isolates as a specific strain (ST11) with six clusters. Notably, all harbored mutations in the *mgrB* gene, potentially acquired through horizontal or clonal transmission.

Moving toward the treatment side, Balkrishna et al. elaborated on the potential of the medicinal plant-derived compounds as a promising adjunct therapy for tuberculosis. The authors investigated the antimycobacterial activity of the medicinal plant *Solanum virginianum* (SVE). SVE extracts were able to inhibit the growth and viability of the *Mycobacterium smegmatis*, a soil-dwelling bacterium of the *Mycobacterium* genus. SVE treatment perturbs the stress tolerance capacity of the *M. smegmatis*, eventually causing an overarching effect on the bacterial cell wall. Importantly, these cell membrane defects lead to enhanced bioavailability or accessibility to the anti-mycobacterial drug, isoniazid. This research study provides a novel plant-based drug molecule that has potential for the adjunct anti-tubercular therapy and can counteract the emerging strains of AMR.

Further, Rodjun et al. conducted a simulation study to optimize dosage regimens for colistin and sitafloxacin, both individually and in combination, against highly resistant *A. baumannii*. The study aimed to identify optimal drug combinations and dosages to effectively treat these infections. By simulating various dosing scenarios and patient conditions, the study determined that a suboptimal colistin dose combined with a supraoptimal sitafloxacin dose could be a promising approach. This combination showed potential for improved treatment outcomes while potentially reducing the risk of adverse effects associated with higher colistin doses. The study highlights the importance of optimizing antibiotic therapy in the face of increasing AMR.

Review article by Shi et al. summarizes the mechanisms responsible for antibiotic resistance in *A. baumannii*, with a particular focus on tigecycline and polymyxin resistance. They highlighted the bacterium's adaptability, showcasing its mechanism of multiple resistance strategies, including antibiotic inactivation, target site modification, altered drug permeability, and other defense mechanisms.

In another review Bhat et al. explored the structure and functions of integrons and their mechanisms. Integrons are natural cloning vectors that facilitate the spread of AMR genes among microbial species. Horizontal gene transfer between different trophic groups is responsible for the dissemination of antibiotic resistance in humans. Additionally, integrons serve as genetic markers for estimating AMR. By estimating antibiotic resistance through integrons, we can better understand their role and develop strategies to counteract the resistance mechanisms evolved by microbes.

In a very unique study, Xiong et al. examined the bacterial composition of extended boar semen and identified *Pseudomonas* species as the predominant bacteria. They isolated a specific strain, designated GXZC, belonging to the *Pseudomonas fluorescens* group. This strain was found to negatively impact sperm quality and exhibit resistance to multiple antibiotics. Also, comparative genomic analysis revealed that the GXZC strain possesses intrinsic and acquired resistance genes. These findings highlight a significant challenge in the long-term storage of extended boar semen due to the presence of the *P. fluorescens* strain.

Another interesting article by Abdelsalam Elshenawy et al. shows a descriptive analysis before and during the COVID-19 pandemic to investigate antibiotic prescribing trends for respiratory tract infections (RTIs). The authors examined antibiotic use in 640 patients with RTIs. Results revealed an increased use of amoxicillin-clavulanic acid and azithromycin. Additionally, there was a rise in antibiotic prescriptions from the “Watch” category during the pandemic, emphasizing the need for enhanced antibiotic management practices. The insights gained into antibiotic prescribing patterns will aid in addressing antibiotic management challenges.

Lastly, the editorial section is concluded through a comprehensive review by Sharma et al. which compiles the information related to the current state of AMR amongst several pathogenic bacteria and provides important insights into emerging challenges in AMR, highlighting the complexities involved in addressing this global health threat and the need for multifaceted approaches to combat AMR. Addressing AMR demands a multi-faceted approach, including integrating AMR into the Sustainable Development Goals (SDGs) to reinforce global healthcare commitment to tackling this Research Topic.

Overall, we believe that collection of articles in this Research Topic will empower the scientific and clinical communities to make informed decisions, guiding the development of more effective research approaches and treatment strategies. By shedding new light on these critical areas, we hope to accelerate progress and improve outcomes for patients worldwide.

